# Sex-dependent influence of maternal predictors on fetal anthropometry in pregnancies with gestational diabetes mellitus

**DOI:** 10.1186/s12884-022-04767-z

**Published:** 2022-06-01

**Authors:** Maria-Christina Antoniou, Leah Gilbert, Justine Gross, Jean-Benoît Rossel, Céline Julie Fischer Fumeaux, Yvan Vial, Jardena Jacqueline Puder

**Affiliations:** 1 Pediatric Service, Department Woman Mother Child, University Hospital of Lausanne, 1011 Lausanne, Switzerland; 2 Obstetric Service, Department Woman Mother Child, University Hospital of Lausanne, 1011 Lausanne, Switzerland; 3 Service of Endocrinology, Diabetes and Metabolism, University Hospital of Lausanne, 1011 Lausanne, Switzerland; 4 Center for Primary Care and Public Health (Unisanté), University of Lausanne, Lausanne, Switzerland; 5 Clinic of Neonatology, Department Woman Mother Child, University Hospital of Lausanne, 1011 Lausanne, Switzerland

**Keywords:** Gestational diabetes, Fetal ultrasound, Fetal anthropometry, Fetal sex, Risk stratification

## Abstract

**Background:**

Third trimester fetal anthropometric parameters are known to predict neonatal complications. A better understanding of predictors of adverse fetal parameters might help to personalize the use and frequency of fetal ultrasound. The objectives of this study were:

(a) to evaluate the utility of maternal sociodemographic, anthropometric and metabolic predictors to predict 3^rd^ trimester fetal anthropometric parameters in women with gestational diabetes mellitus (GDM),

(b) to assess whether the impact of these maternal predictors is fetal sex-dependent, and

(c) to provide a risk stratification for markers of fetal overgrowth (fetal weight centile (FWC) and fetal abdominal circumference centile (FACC) depending on prepregnancy BMI and gestational weight gain (GWG) until the 1^st^ GDM visit.

**Methods:**

This prospective study included 189 women with GDM. Maternal predictors were age, ethnicity, prepregnancy BMI, GWG and excessive weight gain until the 1^st^ GDM visit, fasting, 1-hour and 2-hour blood glucose oral glucose tolerance test values, HbA1c at the 1^st^ visit and medical treatment requirement. Fetal outcomes included FWC, FWC >90% and <10%, FACC, FACC >90% and <10%, at 29 0/7 to 35 6/7 weeks of gestational age. We performed univariate and multivariate regression analyses and probability analyses.

**Results:**

In multivariate analyses, prepregnancy BMI was associated with FWC, FWC > 90% and FACC. GWG until the 1^st^ GDM visit was associated with FWC, FACC and FACC > 90% *(all p ≤ 0.045)*. Other maternal parameters were not significantly associated with fetal anthropometry in multivariate analyses *(all p ≥ 0.054)*. In female fetuses, only GWG was associated with FACC (*p= 0.044*). However, in male fetuses, prepregnancy BMI was associated with FWC, FWC > 90% and FACC and GWG with FWC in multivariate analyses *(all p ≤ 0.030).* In women with a prepregnancy BMI of ≥ 25 kg/m^2^ and a GWG until the 1^st^ GDM visit ≥ 10.3 kg (mean GWG), the risk for FWC > 90% and FACC > 90% was 5.3 and 4 times higher than in their counterparts.

**Conclusions:**

A personalized fetal ultrasound surveillance guided by fetal sex, prepregnancy BMI and GWG may be beneficial in reducing adverse fetal and neonatal outcomes.

**Supplementary Information:**

The online version contains supplementary material available at 10.1186/s12884-022-04767-z.

## Background

Gestational diabetes mellitus (GDM), defined as any degree of glucose intolerance with onset or first recognition during pregnancy, exposes both mothers and their offspring to short and long-term adverse outcomes [1–6]. Among these, large for gestational and macrosomia age are one of the most frequent adverse neonatal outcomes and they also have a more long-term impact [7, 8]. Maternal prepregnancy BMI and gestational weight gain are associated with adverse neonatal outcomes such as LGA [9–13] . Weight gain in excess of the weight gain recommended by the Institute of Medicine [14] significantly increases the risk for LGA, as well as other neonatal complications, including hypoglycemia, polycythemia, low 5-minute Apgar score, meconium aspiration syndrome [15, 16].

A recent analysis of our group has shown that 3^rd^ trimester fetal anthropometric parameters, such as fetal weight centile (FWC), FWC > 90%, fetal abdominal circumference centile (FACC), and FACC > 90% can predict neonatal complications, including small and large for gestational age (SGA, LGA), prematurity, and emergency cesarean section [17].

Only a few studies have investigated the impact of maternal anthropometric and metabolic parameters on fetal anthropometry in late pregnancy; these involved predominantly healthy pregnancies with low GDM incidence. A population-based study by Galjaard et al. [18] showed a positive correlation between gestational weight gain (GWG) and estimated fetal weight (EFW) apparent from the end of the 2^nd^ trimester. Moreover, prepregnancy BMI has also been associated with EFW especially from midpregnancy onward, in predominantly healthy populations [18–20]. In a Korean study population with low GDM incidence (5.1%), maternal age and HbA1c at 24-28 gestational weeks were associated with fetal abdominal overgrowth ratios [21]. In a small cross-sectional study including 19 women with normal glucose tolerance and 12 women with gestational diabetes, estimated fetal weight in late gestation (32-36.6 gestational weeks) was correlated with hepatic glucose production and insulin sensitivity glucose infusion rate at the same timepoint [22].

Male sex has been found to be an independent factor for adverse pregnancy outcomes in predominantly healthy as well as GDM populations [23, 24]. Furthermore, the impact of GDM on fetal abdominal circumference could also be more pronounced in male fetuses, which could mean that this sexual dimorphism starts *in utero* [25]. Thus, Macaulay et al [25] found a positive correlation between GDM diagnosis and fetal abdominal circumference in the whole population, but when stratified by sex, this was only observed in the male fetuses.

Data on the association between maternal characteristics and 3^rd^ trimester fetal anthropometry in pregnancies with GDM are still lacking. Moreover, it is unknown whether these associations follow a sex dimorphism. A personalized follow-up based on maternal characteristics, fetal sex and 3^rd^ trimester anthropometry could possibly lead to a reduction of neonatal complications and long-term adverse outcomes in the offspring.

The objectives of this study were: (a) to evaluate the utility of maternal sociodemographic, anthropometric and metabolic parameters for the prediction of 3^rd^ trimester fetal anthropometric parameters known to be associated with adverse neonatal outcomes in women with GDM, (b) to assess whether the impact of these maternal parameters is fetal sex-dependent and (c) to provide a risk stratification for FWC > 90% and FACC > 90% depending on the prepregnancy BMI and GWG until the 1^st^ GDM visit.

## Methods

This prospective observational study included pregnant women with GDM followed in the Diabetes and Pregnancy Unit in the Centre Hospitalier Universitaire Vaudois (CHUV), Lausanne, Switzerland, between April 2012 and October 2017. Detailed information on the materials and methods have been included in previous publications of our group [12, 17, 26–29] Briefly, all women with GDM who had signed an informed consent were included in this study. Exclusion criteria for the current analysis were: multiple gestation, pregestational diabetes or diabetes diagnosed before 13 weeks of gestation, missing fetal sex and missing fetal ultrasound data between 29 0/7 and 35 6/7 gestational weeks.

GDM was diagnosed according to the International Association of the Diabetes and Pregnancy Study Groups criteria [30], with a 75-g oral glucose tolerance test (oGTT) at 24–28 gestational weeks. The treatment was based on the latest guidelines of the American Diabetes Association [31] and of the Endocrine Society [32]. The patients were followed by a multidisciplinary team specialised in GDM, composed by medical doctors, nurses, and dieticians. At their first clinical appointment, patients received information on GDM, and were instructed on lifestyle adaptations and how to perform a capillary blood glucose test. There were seen a week later by a dietician, who provided them with advice to optimal glycemic control, optimal gestational weight gain and a balanced diet. Women were encouraged to increase physical activity and were offered the possibility to receive physical activity counselling by a physiotherapist, and to participate in GDM physical activity groups.

According to international and local guidelines (Vaud Cantonal Diabetes Program women were asked to check their capillary glucose values 4x/day. If, despite lifestyle changes, glucose values remained above targets, metformin or insulin treatment was introduced, according to guidelines [30–33].

### Maternal predictors and fetal anthropometric outcomes

Maternal predictors included age, ethnicity, prepregnancy body mass index (BMI), GWG until the 1^st^ GDM visit, excessive weight gain (EWG) up to the 1^st^ GDM visit, fasting, 1-h and 2-h blood glucose values during the 75g oGTT at 24–28 weeks of GA, HbA1c at 1^st^ GDM visit, and glucose lowering medical treatment requirement (metformin and/or insulin). Maternal ethnicity was classified, according to official criteria, in Low (Europe, North America) and High Risk (Asia, Central and South America, Africa, Oceania) ethnic groups [34]. Prepregnancy BMI was calculated based on pre-pregnancy weight (retrieved from medical charts or self-reported), and on height measured at the first visit at the GDM clinic, using the formula weight (kg)/(height(m))^2^. GWG was determined as the difference between the last weight measured before delivery and prepregnancy weight. GWG until the 1^st^ GDM visit was determined as the difference between the weight measured at the 1^st^ GDM visit and the prepregnancy weight. EWG at the 1^st^ GDM visit was defined as gestational weight gain exceeding the thresholds established by the Institute of Medicine (IOM) Guidelines 2009 for the respective maternal pre-pregnancy BMI category [14]. HbA1c at the 1^st^ GDM visit was measured using a chemical photometric method (conjugation with boronate; Afinion^®^). Maternal treatment was classified into 2 categories (no treatment, treatment with metformin and/or insulin; where almost all women were treated with insulin).

Fetal anthropometric outcomes consisted of FWC (ranging from 0–100%), FWC > 90%, FWC < 10%, FACC (ranging from 0–100%), FACC > 90% and FACC < 10%. Fetal ultrasounds (one per patient) were performed between 29 0/7 and 35 6/7 weeks of gestation by experienced obstetricians at the CHUV. Estimated FW was calculated using the Hadlock formula [35] and fetal centiles using the Intergrowth 21st fetal size application tool [36].

All maternal and fetal data were retrieved from the CHUV’s patient electronic medical chart.

### Statistical analysis

All data were analysed using Stata/SE 16.0 (StataCorp LLC, TX, USA). The normality of continuous variables was assessed using QQ plots. Continuous variables were normally distributed and described as means and standard deviations (SDs). Binary outcomes were described as N (percentages) (Table 1). These calculations were performed for the total population, as well as for the population stratified by fetal sex, as defined at birth. Comparisons between the female and male subpopulations were initially made using the unpaired t-test for continuous variables and the Fisher’s exact test for binary variables (Table 1). Linear and logistic univariate regression analyses were performed for all women, as well as stratified by fetal sex. In these analyses, fetal outcome was the dependent variable, and analyses were adjusted for fetal gestational age and sex where appropriate (Table 2, Supplementary Tables, Additional files 1 and 2). In female fetuses, for the rare outcome FACC < 10%, two analyses (for ethnicity and excessive weight gain until the 1st GDM visit) was not possible due to the small sample size.

**Table 1:** Descriptive maternal and fetal characteristics

**Maternal Characteristics**	**Total Population**	**Female Fetal Gender**	**Male Fetal Gender**	***p-value***
Number of patients	189	93	96	
Age (years)	32.9 ± 5.4	32.7 ± 5.4	33.0 ± 5.5	0.689
High risk ethnicity (yes; N(%))	81 (43.6%)	41(45.1)	40(42.1)	0.768
Prepregnancy BMI (kg/m^2^)	26.6 ± 5.4	26.4 ± 5.0	26.7 ± 5.8	0.661
Gestational weight gain (kg)	13.3 ± 7.2	13.2 ± 7.0	13.3 ± 7.4	0.958
Gestational weight gain until the 1^st^ GDM visit N(%)	10.5 ± 6.1	10.3 ± 6.7	10.6 ± 6.0	0.751
Excessive weight gain during pregnancy ^a^ (yes; N(%))	88 (46.6%)	45 (48.4)	43 (44.8)	0.663
Excessive weight gain until the 1^st^ GDM visit ^a^ N(%)	145 (76.7%)	72 (77.4)	73 (76.0)	0.864
Fasting oGTT glucose value (mmol/l)	5.3 ± 0.7	5.3 ± 0.7	5.2 ± 0.6	0.271
1-h oGTT glucose value (mmol/l)	10.0 ± 2.1	9.7 ± 2.4	10.2 ± 1.9	0.125
2-h oGTT glucose value (mmol/l)	7.9 ± 2.0	8.1 ± 2.2	7.8 ± 1.9	0.371
Gestational age at the 1^st^ GDM visit (weeks)	28.2 ± 3.0	28.1 ± 2.6	28.3 ± 3.4	0.762
HbA1c at the 1^st^ GDM visit (%)	5.5 ± 0.4	5.5 ± 0.5	5.5 ± 0.4	0.900
(mmol/mol)	37 ± 4.4	37 ± 5.5	37 ± 4.4	0.900
Medical treatment requirement N(%)	104 (58.8)	49 (55.1)	55 (62.5)	0.361
**Fetal Characteristics**				
Gestational age (weeks)	32.8 ± 1.5	32.8 ± 1.5	32.7 ± 1.5	0.674
Fetal weight (gr)	2089± 385	2118 ± 423	2060 ± 345	0.305
Fetal weight centile ^b^ (%)	67.8 ± 21.4	68.5 ± 21.1	67.1 ± 21.7	0.644
Fetal weight centile > 90% ^b^ N(%)	33 (17.5)	19 (20.4)	14 (14.6)	0.340
Fetal weight centile < 10%^b^ N(%)	0 (0)	0 (0)	0(0)	-
Fetal abdominal circumference ^b^ (mm)	289.8 ± 42.9	290.3 ± 37.6	289.4 ± 47.8	0.884
Fetal abdominal circumference centile ^b^ (%)	65.9 ± 29.9	67.4 ± 28.5	64.5 ± 31.3	0.512
Fetal abdominal circumference centile > 90% ^b^ N(%)	54 (28.6)	27 (29.0)	27 (28.1)	1.000
Fetal abdominal circumference centile < 10% ^b^ N(%)	12 (6.4)	4 (4.3)	8 (8.3)	0.373

Descriptive maternal and fetal characteristics (total population and population stratified by fetal gender) and comparisons between maternal and fetal characteristics for female and male fetuses

*Abbreviations*: *BMI* Body mass index, *GDM* Gestational diabetes mellitus, *oGTT* Oral glucose tolerance test, *HbA1c* Glycated haemoglobin

^a^ According to the Institute of Medicine Guidelines 2009 [14]

^b^ Adjusted for gestational age using the Intergrowth 21st fetal size application tool [36]

**Table 2:** Maternal predictors of fetal anthropometric parameters in univariate analyses (total population)

Fetal Anthropometric Parameters	Maternal Predictors	OR / Beta-Coefficient^**c**^	95% CI		*p- value*
Fetal weight centile (%) ^a^	Age (years)	0.36 ^c^	-0.21	0.93	0.209
	Ethnicity (low/high risk)	0.04 ^c^	-6.13	6.20	0.990
	Prepregnancy BMI (kg/m^2^)	0.71 ^**c**^	0.15	1.27	0.013
	Gestational weight gain (kg) until the 1^st^ GDM visit	0.58 ^**c**^	0.09	1.07	0.020
	Excessive weight gain until the 1^st^ GDM visit ^**b**^	9.05 ^**c**^	1.91	16.18	0.013
	Fasting oGTT glucose (mmol/L)	1.80 ^c^	-3.07	6.68	0.467
	1-h oGTT glucose (mmol/L)	1.45 ^c^	-0.23	3.13	0.091
	2-h oGTT glucose (mmol/L)	0.72 ^c^	-1.02	2.47	0.412
	HbA1c at the 1^st^ GDM visit (%/mmol/mol)	5.55 ^c^	-1.36	12.46	0.115
	Medical treatment requirement	7.94 ^**c**^	1.74	14.14	0.012
Fetal weight centile >90 (%) ^a^	Age (years)	1.06	0.98	1.14	0.138
	Ethnicity (low/high risk)	1.25	0.58	2.71	0.573
	Prepregnancy BMI (kg/m^2^)	1.08	1.01	1.15	0.028
	Gestational weight gain (kg) until the 1^st^ GDM visit	1.07	1.00	1.14	0.044
	Excessive weight gain until the 1^st^ GDM visit ^b^	1.86	0.67	5.16	0.234
	Fasting oGTT glucose (mmol/L)	1.32	0.78	2.23	0.295
	1-h oGTT glucose (mmol/L)	1.24	1.01	1.52	0.038
	2-h oGTT glucose (mmol/L)	1.06	0.86	1.30	0.593
	HbA1c at the 1^st^ GDM visit (%/mmol/mol)	1.41	0.62	3.21	0.418
	Medical treatment requirement	1.33	0.60	2.91	0.482
Fetal abdominal circumference centile (%) ^a^	Age (years)	0.59 ^c^	-0.22	1.39	0.151
	Ethnicity (low/high risk)	-2.92 ^c^	-11.67	5.82	0.510
	Prepregnancy BMI (kg/m^2^)	0.91 ^**c**^	0.12	1.70	0.025
	Gestational weight gain (kg) until the 1^st^ GDM visit	0.87 ^**c**^	0.17	1.56	0.015
	Excessive weight gain until the 1^st^ GDM visit ^**b**^	10.27 ^**c**^	0.13	20.41	0.047
	Fasting oGTT glucose (mmol/L)	1.86 ^c^	-5.03	8.75	0.595
	1-h oGTT glucose (mmol/L)	2.46 ^**c**^	0.07	4.85	0.044
	2-h oGTT glucose (mmol/L)	0.53 ^c^	-1.95	3.01	0.672
	HbA1c at the 1^st^ GDM visit (%/mmol/mol)	8.41 ^c^	-1.42	18.23	0.093
	Medical treatment requirement	9.94 ^**c**^	1.03	18.85	0.029
Fetal abdominal circumference centile >90 (%) ^a^	Age (years)	1.03	0.97	1.10	0.292
	Ethnicity (low/high risk)	0.93	0.49	1.78	0.827
	Prepregnancy BMI (kg/m^2^)	1.05	0.99	1.12	0.074
	Gestational weight gain (kg) until the 1^st^ GDM visit	1.08	1.02	1.15	0.006
	Excessive weight gain until the 1^st^ GDM visit ^b^	2.09	0.90	4.85	0.086
	Fasting oGTT glucose (mmol/L)	1.28	0.80	2.04	0.307
	1-h oGTT glucose (mmol/L)	1.21	1.01	1.45	0.036
	2-h oGTT glucose (mmol/L)	1.00	0.84	1.20	0.963
	HbA1c at the 1^st^ GDM visit (%/mmol/mol)	1.60	0.79	3.27	0.193
	Medical treatment requirement	1.96	0.99	3.90	0.054
Fetal abdominal circumference centile <10 (%) ^a^	Age (years)	0.97	0.87	1.08	0.600
	Ethnicity (low/high risk)	1.35	0.42	4.37	0.617
	Prepregnancy BMI (kg/m^2^)	0.93	0.82	1.05	0.232
	Gestational weight gain (kg) until the 1^st^ GDM visit	1.01	0.91	1.11	0.901
	Excessive weight gain until the 1^st^ GDM visit ^b^	1.58	0.33	7.53	0.566
	Fasting oGTT glucose (mmol/L)	0.71	0.24	2.08	0.527
	1-h oGTT glucose (mmol/L)	0.77	0.54	1.11	0.166
	2-h oGTT glucose (mmol/L)	0.82	0.56	1.20	0.310
	HbA1c at the 1^st^ GDM visit (%/mmol/mol)	0.30	0.07	1.39	0.124
	Medical treatment requirement	0.85	0.22	3.31	0.819

Linear and logistic regression analyses, adjusted for neonatal gender and gestational age

*Abbreviations*: *OR* Odds ratio, *CI* Confidence interval, *BMI* Body mass index, *GDM* Gestational diabetes mellitus, *oGTT* Oral glucose tolerance test, *HbA1c* Glycated hemoglobin

^a^ Adjusted for gestational age using the Intergrowth 21st fetal size application tool [36]

^b^ According to the Institute of Medicine Guidelines 2009 [14]

^c^ This value corresponds to a beta-coefficient

Maternal predictors with a *p*-value < 0.05 in univariate analysis were included in a stepwise multiple regression analysis model. These analyses were also adjusted for fetal gestational age and gender where appropriate. These analyses were performed in order to identify the most important maternal predictors of fetal anthropometric parameters associated with adverse neonatal outcomes in previous studies (Tables 3 and 4). We tested for collinearity and collinearity index was less than 0.6 for all predictors.

**Table 3:** Maternal predictors of fetal anthropometric parameters in multivariate analyses (total population)

Fetal Anthropometric Parameters	Maternal Predictors	OR / Beta-Coefficient ^**c**^	95% CI		*p-value*
Fetal weight centile (%) ^a^	Prepregnancy BMI (kg/m^2^)	0.66 ^c^	0.10	1.23	0.022
	Gestational weight gain (kg) until the 1^st^ GDM visit	0.62 ^c^	0.12	1.12	0.016
	Excessive weight gain until the 1^st^ GDM visit ^b^				0.934
	Medical treatment requirement				0.057
Fetal weight centile >90 (%) ^a^	Prepregnancy BMI (kg/m^2^)	1.09	1.01	1.18	0.019
	Gestational weight gain (kg) until the 1^st^ GDM visit				0.128
	1-h oGTT glucose (mmol/L)				0.054
Fetal abdominal circumference centile (%) ^a^	Prepregnancy BMI (kg/m^2^)	0.93 ^c^	0.02	1.84	0.045
	Gestational weight gain (kg) until the 1^st^ GDM visit	0.98 ^c^	0.12	1.83	0.025
	Excessive weight gain until the 1^st^ GDM visit ^b^				0.619
	1-h oGTT glucose (mmol/L)				0.168
	Medical treatment requirement				0.288
Fetal abdominal circumference centile >90 (%) ^a^	Gestational weight gain (kg) until the 1^st^ GDM visit	1.09	1.02	1.17	0.009
	1-h oGTT glucose (mmol/L)				0.077

Stepwise multiple regression analyses, adjusted for neonatal gender and gestational age

Abbreviations: OR odds ratio, CI confidence interval, BMI body mass index, GDM gestational diabetes mellitus, oGTT oral glucose tolerance test

^a^ For gestational age using the Intergrowth 21st fetal size application tool [36]

^b^ According to the Institute of Medicine Guidelines 2009 [14]

^c^ This value corresponds to a beta-coefficient

**Table 4:** Maternal predictors of male fetal anthropometric parameters in multivariate analyses

Fetal Anthropometric Parameters	Maternal Predictors	OR / Beta-Coefficient ^**b**^	95% CI		*p- value*
Fetal weight centile (%) ^a^	Prepregnancy BMI (kg/m^2^)	1.27 ^b^	0.57	1.97	0.001
	Gestational weight gain (kg) until the 1^st^ GDM visit	0.98 ^b^	0.26	1.70	0.008
Fetal weight centile >90 (%) ^a^	Prepregnancy BMI (kg/m^2^)	1.14	1.03	1.25	0.009
	Gestational weight gain (kg) until the 1^st^ GDM visit	1.07	1.00	1.14	0.128
Fetal abdominal circumference centile (%) ^a^	Prepregnancy BMI (kg/m^2^)	1.22 ^b^	0.12	2.31	0.030
	Medical treatment requirement				0.079
Fetal abdominal circumference centile >90 (%) ^a^	Gestational weight gain (kg) until the 1^st^ GDM visit	1.09	1.00	1.19	0.050

Stepwise multiple regression analyses, adjusted for gestational age

*Abbreviations*: *OR* Odds ratio, *CI* Confidence interval, *BMI* Body mass index, *GDM* Gestational diabetes mellitus

^a^ For gestational age using the Intergrowth 21st fetal size application tool [36]

^b^ This value corresponds to a beta-coefficient

Probability analyses according to logistic regression models were used to evaluate the risk of FWC > 90% and FACC > 90% based on two maternal parameters that are easily available at the 1st GDM booking and turned out to be very predictive: prepregnancy BMI and GWG until the 1^st^ GDM visit > 10.35 kg, which corresponds to the median value at this timepoint (Table 5). We used GWG as it was the most relevant predictor for fetal outcomes, in addition to prepregnancy BMI. However, we also tested the same analyses using the presence or not of EWG based on the IOM criteria instead of the median GWG until the 1^st^ GDM visit (Additional file 3, Table A3).

**Table 5:** Risk stratification for fetal overgrowth based on prepregnancy BMI and initial GWG

**Prepregnancy BMI (kg/m**^**2**^**)**	**GWG at the 1**^**st**^** GDM visit (kg)**	**Fetal weight centile >90% **^**a**^	**Fetal abdominal circumference centile > 90% **^**a**^
< 25	< 10.3	6%	12%
< 25	≥ 10.3	18%	30%
≥ 25	< 10.3	12%	23%
≥ 25	≥ 10.3	32%	48%

Probability analyses using logistic regression models to assed risk for fetal overgrowth at the 3^rd^ trimester

*Abbreviations*: *BMI* Body mass index, *GWG* Gestational weight gain, *GDM* Gestational diabetes mellitus

^a^ adjusted for gestational age using the Intergrowth 21st fetal size application tool [36]

For all analyses, beta-coefficients (for continuous outcomes such as FWC and FACC) and adjusted odds ratios (aORs-for binary outcome, including FWC > 90%, FACC > 90% and <10%) are reported along with their 95% confidence intervals (CIs), and statistical significance was set at 0.05.

## Results

The initial population included 831 adult women with gestational diabetes, of whom 9 were excluded because they participated in an intervention trial and 111 because they refused to participate in the study. Of the 711 women who agreed to participate and signed informed consent, the following were excluded: 142 due to multiple gestation and/or missing sex at birth, 5 because the diagnosis of GDM was done before 13 weeks of gestation, raising the suspicion of pre-existent diabetes and 375 because of missing fetal ultrasound data between 29 0/7 and 35 6/7 gestational weeks (as there were done in outside private practices). Thus, 189 women were included in the final analysis.

### Maternal sociodemographic, anthropometric and metabolic characteristics and fetal anthropometric parameters

Detailed information on the maternal characteristics and fetal anthropometric parameters of the total population, as well as the population stratified by fetal sex, are displayed in Table 1. No significant differences in the maternal characteristics and fetal anthropometry were found when stratifying the population by fetal sex.

### Associations between maternal predictors and fetal anthropometric parameters

#### Total population

The results of the univariate analyses are shown in Table 2: Prepregnancy BMI showed a significant association with FWC, FWC > 90% and FACC, and 1-hour-oGTT glucose with FWC > 90%, FACC and FACC > 90%. GWG until the 1^st^ visit at the GDM clinic predicted FWC, FWC > 90%, FACC and FACC > 90%. Excessive weight gain until the 1^st^ visit was associated with FWC, and FACC. Maternal medical treatment requirement (metformin and/or insulin) was associated with FWC and FACC (all *p* ≤ 0.047). Maternal age, ethnicity, fasting-oGTT glucose, 2-hour-oGTT glucose and HbA1c at the first and last GDM visit did not show any association with fetal outcomes.

The results of the multivariate analyses are shown in Table 3: GWG until the 1^st^ visit at the GDM clinic predicted FWC, FACC and FACC > 90% and prepregnancy BMI showed a significant association with FWC, FWC > 90% and FACC (all *p* ≤ 0.045). A marginal association was found between maternal medical treatment requirement and FWC (*p=* 0.057) as well as 1-hour-oGTT glucose and FWC > 90% (*p=* 0.054).

#### Population stratified by fetal gender

In female fetuses, only GWG until the 1^st^ GDM visit showed a signification association with FACC (both in the univariate and thus also in the multivariate analyses; *p*= 0.040, see Additional file 1, Table A1). Otherwise, no other association was found between maternal predictors and female fetal anthropometric parameters.

In male fetuses, however, prepregnancy BMI, GWG until the 1^st^ GDM visit and maternal medical treatment requirement showed a significant association with one or more fetal anthropometric parameters. More precisely, prepregnancy BMI predicted FWC, FWC > 90% and FACC (all *p* ≤ 0.009 see Additional file 2, Table A2). GWG until the 1^st^ GDM visit showed a significant association with FWC and a marginally significant association with FACC > 90% and the need for maternal treatment a significant association with FACC (all *p* ≤ 0.050 see see Additional file 2, Table A2). In multivariate analyses for the US outcomes of males fetuses, prepregnancy BMI was associated with FWC, FWC > 90% and FACC and GWG until the 1^st^ GDM visit with FWC (all *p* ≤ 0.030 see Table 4).

#### Risk stratification

Probability analyses using logistic regression models were performed to provide a risk stratification for FWC > 90%, and FACC > 90%, according to the prepregnancy BMI (< 25 vs ≥ 25 kg/m^2^) and GWG until the 1^st^ GDM visit (< 10.3 vs > 10.3 kg corresponding to the median GWG until his timepoint). In the lowest risk group (BMI < 25 kg/m^2^ and GWG until the 1^st^ GDM visit < 10.3 kg), the probability for FWC > 90% was 6% and for FACC > 90% was 12%, whereas this risk was 5.3 times higher (32%) for FWC > 90% and 4.0 times higher (48%) for FACC > 90% in the highest risk group (BMI ≥ 25 kg/m^2^ and GWG until the 1^st^ GDM visit ≥ 10.3kg) (Table 5). Results were similar when using EWG instead of GWG (see Additional file 3, Table A3).

## Discussion

This prospective cohort of 189 singleton women with GDM showed that two maternal anthropometric parameters, prepregnancy BMI and GWG until the 1^st^ visit at the GDM clinic were the only independent predictors of 3^rd^ trimester fetal anthropometry Thus, these two parameters could serve to determine the frequency of US monitoring in this metabolically high-risk population of women with GDM. When stratifying the population by fetal sex, prepregnancy BMI and/or GWG until the 1^st^ GDM visit predicted FWC, FWC > 90%, and FACC, in multivariate analyses in male fetuses. In female fetuses, however, only GWG until the 1^st^ GDM visit was predictive and predicted only FACC. A risk stratification model showed that in overweight or obese women with a GWG until the 1^st^ GDM visit above the median (≥10.3 kg at 28.2 weeks of GA), the risk for FWC > 90% and FACC > 90% was 4-5 times higher than in normal-weight women with a GWG below the median. For the total population, prepregnancy BMI predicted FWC, FWC > 90% and FACC and GWG until the 1^st^ visit at the GDM clinic predicted FWC, FACC and FACC > 90% in multivariate analyses. In addition, in univariate analysis, 1-h glucose values after oGTT and the need for medical treatment also had an impact on fetal anthropometry, but not the other tested sociodemographic or metabolic predictors. The impact of various maternal parameters on 3^rd^ trimester fetal anthropometry in pregnancies complicated with GDM has been previously poorly studied; most of the existing data have been obtained from healthy pregnancies with low GDM or unspecified GDM incidence, while for pregnancies with GDM, data are based on neonatal, but not fetal anthropometry [12, 18, 21, 37].

In population-based mostly healthy pregnancies, GWG has been positively associated with estimated FW and FAC [18, 38]. Similarly, prepregnancy BMI has been significantly associated with estimated FW from midprepregnancy onwards [20]. Moreover, a cohort study with 11.2% of women with GDM, described a positive association between GDM status and FAC [25] . However, in this same study, maternal BMI and weight change per week had a direct effect on FAC that was independent of GDM status. A prospective cohort study in a population with 4.2% GDM incidence, demonstrated that obese women with GDM had an increased risk of FACC >90% [39].

In a study investigating fetal growth in pregnancies with Type 1, Type 2 diabetes, and GDM vs controls, the diabetes category influenced the AC growth trajectory, but ethnicity, maternal prepregnancy weight and BMI did not [40]. Moreover, in a small study of 31 women including also women with GDM (present in 39% of the population), insulin resistance, but not maternal age or weight gain was correlated to estimated fetal weight. Differences between these studies and ours are that we used a larger and homogenous cohort of women with GDM. Thus, the impact of BMI and weight gain in the cited studies might have been diluted by differences in glucose control. Furthermore, in contrast to the first study, we used fetal anthropometry at a given time point and not the trajectory [22].

In our study, excessive weight gain until the 1^st^ GDM visit showed a significant association with FWC and FACC in univariate but not in multivariate analyses. That could mean that the GWG and not EWG according to the IOM 2009 criteria, is the most relevant parameter and could question the relevance of the IOM cut-offs for this population [41]. This finding is in accordance with a previous study of our group which found that in the presence of GWG, EWG was not associated with adverse neonatal outcomes, suggesting again the superiority of absolute GWG to predict these outcomes [12]. To the best of our knowledge, the effect of EWG on fetal anthropometry has not been studied previously.

Male offspring are known to have higher perinatal risks, such as preterm birth, cord prolapse, cesarean section, lower Apgar score at 1 minute, and higher fetal anthropometry measures [23, 24]. In a general healthy Caucasian population, higher abdominal and head circumference, were documented in male vs female fetuses throughout gestation [42]. We therefore stratified our analyses by sex: when doing this, the association between maternal parameters and fetal anthropometry was predominantly observed among male fetuses. To the best of our knowledge, this is the first study investigating the presence of a sexual dimorphism in the effect of maternal predictors on 3rd trimester anthropometry in pregnancies with GDM. Most of the existing studies in populations with GDM have investigated the effect of the fetal sex on pregnancy outcomes, as well as metabolic complications in neonates or later on in life. Fetal growth in populations with GDM has previously been studied without taking fetal sex into account. In the general population, fetal growth is also monitored without consideration for fetal sex, even though some sex-specific growth charts exist [42, 43]. Regarding neonatal complications, previous population-based studies, have shown that male sex was an independent risk factor for adverse perinatal outcomes, including preterm birth, lower Apgar scores, macrosomia, [23, 44–46] as well as higher birthweight, and lower arterial pH [42, 46]. A higher risk for pregnancy complications has been also found for women carrying male fetuses, with higher incidence of gestational diabetes, caesarean section, cord prolapse and nuchal cords [23]. A recent study in a population with GDM found higher birthweight, and fat mass at birth among male vs female neonates, and a more frequent need for insulin treatment in their mothers [24]. Hu et al., [46] found a higher risk for neonatal infection, acute respiratory disorders and abnormal neonatal central nervous system development in male vs female fetuses in pregnancies with GDM. Lastly, a higher BMI and risk for obesity during childhood was observed among male but not female offspring from GDM exposed pregnancies [47].

Finally, we performed probability analyses using logistic regression models in order to provide a risk stratification for two markers of 3rd trimester fetal overgrowth, according to the prepregnancy BMI and the GWG until the 1st GDM visit. FWC > 90%, and FACC > 90% have been found to be powerful predictors of adverse neonatal outcomes [17], and prepregnancy BMI and GWG until the 1^st^ GDM visit are easily available at the 1^st^ visit. In the highest risk group (prepregnancy BMI of ≥ 25 kg/m^2^ and GWG until the 1st GDM visit ≥ 10.3 kg), the risk for FWC > 90% and FACC > 90% was 4-5 times higher compared to the lowest risk group. Interestingly, although the proposed ideal weight gain for normal-weight women for the entire pregnancy is 11.3-16 kg [48], gaining more than 10.3 kg up to the first GDM visit in this group was also associated with 2.5-3 times increase in fetal overgrowth. Of note, none of the fetal US showed a FWC < 10% and for FACC < 10% this was only observed in 6.4% of the total population. Using EWG instead of GWG for the risk stratification provided similar results. To the best of our knowledge, this is the first study providing a risk stratification model for 3^rd^ trimester fetal overgrowth, according to maternal anthropometric parameters.

Based on our results, aiming to reduce GWG starting early in pregnancy seems crucial, as more pronounced weight gain in this time period may be associated with the risk of fetal overgrowth but also offspring complications [12]. Similarly, achieving weight loss in overweight and obese women before pregnancy may help reduce fetal overgrowth and neonatal complications. In the long-term, a personalized follow-up may be offered to women with GDM based on their prepregnancy BMI, their gestational weight gain, as well as anthropometric fetal parameters and fetal gender.

The strengths of our study encompass its original findings in the multivariate and stratified analyses as well as its prospective nature, as it contains thorough information on maternal and fetal parameters of interest. Some limitations may nevertheless be mentioned. For the rare outcome FACC < 10%, two analyses were not possible due to the small sample size. Moreover, we included only 3rd and not 2nd trimester fetal anthropometric data due to the limited number of patients followed at our hospital before the diagnosis of GDM. In order to ensure data quality, all fetal anthropometric data included in the analyses were exclusively obtained from ultrasounds performed in our CHUV tertiary hospital, by equally experienced gynecologists, using the same methodology. Lastly, we included in our analyses 189 patients of the initial population of 831 women. Although metabolic parameters were similar in our selected cohort compared to the initial population of 711 women who consented, we cannot be completely sure that this is a representative sample.

## Conclusions

This study showed that in women with GDM, prepregnancy BMI and GWG until the 1st visit at the GDM clinic are the only significant predictors of 3rd trimester fetal anthropometric parameters. The influence of these maternal parameters presents a sex dimorphism, affecting predominantly male fetuses. Compared to normal-weight women with a GWG less than 10.3 kg up to 28.2 weeks of GA, fetal overgrowth is 2.5-3 times higher in those who gain more than 10.3 kg in this time period and even 4-5 times higher in overweight or obese women with a GWG of 10.3 kg or more. A personalized follow-up guided by the fetal sex and anthropometry as well as maternal metabolic control may be useful in women with high GWG until the end of the second trimester and/or high prepregnancy BMI.

## Supplementary Information

**Additional file 1.**

**Additional file 2.**

**Additional file 3.**

## Data Availability

The datasets generated and/or analysed during the current study are not publicly available, as many of them are still being cleaned and worked on but are available from the corresponding author on reasonable request.

## Abbreviations

BMI: Body mass index
CHUV: Centre Hospitalier Universitaire Vaudois
CI: Confidence interval
EFW: Estimated fetal weight
EGW: Excessive weight gain
FACC: Fetal abdominal circumference centile
FWC: Fetal weight centile
GDM: Gestational diabetes mellitus
GWG: Gestational weight gain
HbA1c: Glycated hemoglobin
IOM: Institute of medicine
LGA: Large for gestational age
oGTT: Oral glucose tolerance test
OR: Odds ratio
SGA: Small for gestational age; US: ultrasound
