# Supplementary material for: Sex-dependent influence of maternal predictors on fetal anthropometry in pregnancies with gestational diabetes mellitus
Source: BMC Pregnancy Childbirth. 2022 Jun 1;22:460. doi: 10.1186/s12884-022-04767-z (PMC9158189; doi:10.1186/s12884-022-04767-z)
Supplement: Supplementary file 1 — Additional file 1. [file 12884_2022_4767_MOESM1_ESM.docx]

Additional file 1

**Table A1.** Maternal predictors of female fetal anthropometric parameters in univariate analyses

| Fetal Anthropometric Parameters | Maternal Predictors | OR/  Beta- Coefficient^††^ | Standard Error | 95% CI | | *p- value* |
| --- | --- | --- | --- | --- | --- | --- |
| Fetal weight centile (%) ^*^ | Age (years) | 0.54^††^ | 0.41 | -0.26 | 1.35 | 0.185 |
|  | Ethnicity (low/high risk) | -1.84^††^ | 4.39 | -10.56 | 6.88 | 0.675 |
|  | Prepregnancy BMI (kg/m^2^) | 0.04^††^ | 0.44 | -0.84 | 0.91 | 0.936 |
|  | Gestational weight gain (kg) | 0.52^††^ | 0.31 | -0.10 | 1.15 | 0.100 |
|  | Gestational weight gain (kg) until the 1^st^ GDM visit | 0.46^††^ | 0.33 | -0.20 | 1.12 | 0.171 |
|  | Excessive weight gain until the 1^st^ GDM visit^†^ | 8.67^††^ | 5.23 | -1.73 | 19.06 | 0.101 |
|  | Fasting oGTT glucose (mmol/L) | 3.14^††^ | 3.07 | -2.96 | 9.25 | 0.309 |
|  | 1-h oGTT glucose (mmol/L) | 1.14^††^ | 1.19 | -1.23 | 3.52 | 0.339 |
|  | 2-h oGTT glucose (mmol/L) | 1.51^††^ | 1.16 | -0.81 | 3.82 | 0.198 |
|  | HbA1c at the 1st GDM visit (%/mmol/mol) | 6.45^††^ | 4.80 | -3.08 | 15.99 | 0.182 |
|  | Medical treatment requirement | 3.76^††^ | 4.64 | -5.46 | 12.99 | 0.420 |
| Fetal weight centile >90 (%) ^*^ | Age (years) | 1.05 | 0.05 | 1.00 | 1.16 | 0.290 |
|  | Ethnicity (low/high risk) | 1.28 | 0.68 | 0.46 | 3.60 | 0.638 |
|  | Prepregnancy BMI (kg/m^2^) | 1.02 | 0.05 | 0.92 | 1.12 | 0.761 |
|  | Gestational weight gain (kg) | 1.07 | 0.05 | 0.99 | 1.17 | 0.103 |
|  | Gestational weight gain (kg) until the 1^st^ GDM visit | 1.06 | 0.05 | 0.98 | 1.16 | 0.144 |
|  | Excessive weight gain until the 1^st^ GDM visit^†^ | 2.94 | 2.33 | 0.62 | 13.91 | 0.175 |
|  | Fasting oGTT glucose (mmol/L) | 1.34 | 0.42 | 0.73 | 2.48 | 0.349 |
|  | 1-h oGTT glucose (mmol/L) | 1.22 | 1.16 | 0.95 | 1.56 | 0.127 |
|  | 2-h oGTT glucose (mmol/L) | 1.13 | 0.15 | 0.87 | 1.48 | 0.355 |
|  | HbA1c at the 1st GDM visit (%/mmol/mol) | 1.25 | 0.68 | 0.43 | 3.61 | 0.683 |
|  | Medical treatment requirement | 0.88 | 0.46 | 0.32 | 2.44 | 0.811 |
| Fetal abdominal circumference centile (%) ^*^ | Age (years) | 0.48^††^ | 0.55 | -0.62 | 1.58 | 0.389 |
|  | Ethnicity (low/high risk) | -9.28^††^ | 5.93 | -21.06 | 2.49 | 0.121 |
|  | Prepregnancy BMI (kg/m^2^) | 0.12^††^ | 0.60 | -1.07 | 1.31 | 0.839 |
|  | Gestational weight gain (kg) | 1.15^††^ | 0.43 | 0.29 | 2.01 | 0.010 |
|  | Gestational weight gain (kg) until the 1^st^ GDM visit | 0.93^††^ | 0.44 | 0.04 | 1.81 | 0.040 |
|  | Excessive weight gain until the 1^st^ GDM visit^†^ | 12.59^††^ | 7.07 | -1.46 | 26.64 | 0.078 |
|  | Fasting oGTT glucose (mmol/L) | 2.98^††^ | 4.18 | -5.32 | 11.28 | 0.478 |
|  | 1-h oGTT glucose (mmol/L) | 2.43^††^ | 1.61 | -0.78 | 5.64 | 0.136 |
|  | 2-h oGTT glucose (mmol/L) | 1.61^††^ | 1.61 | -1.60 | 4.82 | 0.320 |
|  | HbA1c at the 1st GDM visit (%/mmol/mol) | 9.74^††^ | 6.50 | -3.17 | 22.66 | 0.137 |
|  | Medical treatment requirement | 5.41^††^ | 6.34 | -7.19 | 18.01 | 0.396 |
| Fetal abdominal circumference centile >90 (%) ^*^ | Age (years) | 1.01 | 0.04 | 0.93 | 1.10 | 0.782 |
|  | Ethnicity (low/high risk) | 0.86 | 0.40 | 0.34 | 2.14 | 0.739 |
|  | Prepregnancy BMI (kg/m^2^) | 1.05 | 0.05 | 0.96 | 1.15 | 0.243 |
|  | Gestational weight gain (kg) | 1.08 | 0.04 | 1.00 | 1.17 | 0.055 |
|  | Gestational weight gain (kg) until the 1^st^ GDM visit | 1.08 | 0.04 | 1.00 | 1.16 | 0.057 |
|  | Excessive weight gain until the 1^st^ GDM visit^†^ | 1.99 | 1.22 | 0.60 | 6.60 | 0.258 |
|  | Fasting oGTT glucose (mmol/L) | 1.50 | 0.45 | 0.83 | 2.71 | 0.179 |
|  | 1-h oGTT glucose (mmol/L) | 1.25 | 0.16 | 0.97 | 1.60 | 0.081 |
|  | 2-h oGTT glucose (mmol/L) | 1.15 | 1.15 | 0.90 | 1.47 | 0.271 |
|  | HbA1c at the 1st GDM visit (%/mmol/mol) | 1.73 | 0.84 | 0.67 | 4.47 | 0.258 |
|  | Medical treatment requirement | 1.45 | 0.69 | 0.57 | 3.69 | 0.431 |
| Fetal abdominal circumference centile <10 (%) ^*^ | Age (years) | 1.04 | 0.10 | 0.86 | 1.26 | 0.690 |
|  | Ethnicity (low/high risk) | - ^‡^ | - ^‡^ | - ^‡^ | - ^‡^ | - ^‡^ |
|  | Prepregnancy BMI (kg/m^2^) | 0.98 | 0.10 | 0.80 | 1.21 | 0.858 |
|  | Gestational weight gain (kg) | 0.92 | 0.06 | 0.80 | 1.06 | 0.240 |
|  | Gestational weight gain (kg) until the 1^st^ GDM visit | 1.00 | 0.08 | 0.86 | 1.16 | 0.993 |
|  | Excessive weight gain until the 1^st^ GDM visit^†^ | - ^‡^ | - ^‡^ | - ^‡^ | - ^‡^ | - ^‡^ |
|  | Fasting oGTT glucose (mmol/L) | 0.56 | 0.54 | 0.08 | 3.70 | 0.546 |
|  | 1-h oGTT glucose (mmol/L) | 0.82 | 0.22 | 0.49 | 1.38 | 0.460 |
|  | 2-h oGTT glucose (mmol/L) | 0.73 | 0.23 | 0.39 | 1.37 | 0.326 |
|  | HbA1c at the 1st GDM visit (%/mmol/mol) | 0.17 | 0.26 | 0.01 | 3.28 | 0.242 |
|  | Medical treatment requirement | 2.54 | 2.99 | 0.25 | 25.45 | 0.427 |

Linear and logistic regression analyses, adjusted for gestational age

Abbreviations: OR odds ratio, CI confidence interval, BMI body mass index, GDM gestational diabetes mellitus, oGTT oral glucose tolerance test, HbA1c glycated hemoglobin.

* adjusted for gestational age using the Intergrowth 21st fetal size application tool [30]

† according to the Institute of Medicine Guidelines 2009 [28]

†† this value corresponds to a beta-coefficient

‡ statistical analysis not possible due to the small amount of outcomes
