# Supplementary material for: Sex-dependent influence of maternal predictors on fetal anthropometry in pregnancies with gestational diabetes mellitus
Source: BMC Pregnancy Childbirth. 2022 Jun 1;22:460. doi: 10.1186/s12884-022-04767-z (PMC9158189; doi:10.1186/s12884-022-04767-z)
Supplement: Supplementary file 2 — Additional file 2. [file 12884_2022_4767_MOESM2_ESM.docx]

**Additional file 2**

**Table A2.** Maternal predictors of male fetal anthropometric parameters in univariate analyses

| Fetal Anthropometric Parameters | Maternal Predictors | OR/  Beta-Coefficient^††^ | Standard Error | 95% CI | *p- value* |  |  |
| --- | --- | --- | --- | --- | --- | --- | --- |
| Fetal weight centile (%) ^*^ | Age (years) | 0.18^††^ | 0.42 | -0.65 | 1.01 | | 0.662 |
|  | Ethnicity (low/high risk) | 1.85^††^ | 4.52 | -7.12 | 10.82 | | 0.683 |
|  | Prepregnancy BMI (kg/m^2^) | 1.23^††^ | 0.36 | 0.52 | 1.95 | | 0.001 |
|  | Gestational weight gain (kg) | 0.51^††^ | 0.31 | -0.11 | 1.14 | | 0.105 |
|  | Gestational weight gain (kg) until the 1^st^ GDM visit | 0.76^††^ | 0.38 | 0.01 | 1.51 | | 0.048 |
|  | Excessive weight gain until the 1^st^ GDM visit^†^ | 9.62^††^ | 5.08 | -0.47 | 19.72 | | 0.062 |
|  | Fasting oGTT glucose (mmol/L) | -0.63^††^ | 4.18 | -8.93 | 7.67 | | 0.880 |
|  | 1-h oGTT glucose (mmol/L) | 1.79^††^ | 1.25 | -0.71 | 4.29 | | 0.158 |
|  | 2-h oGTT glucose (mmol/L) | -0.32^††^ | 1.35 | -3.01 | 2.38 | | 0.815 |
|  | HbA1c at the 1st GDM visit (%/mmol/mol) | 4.45^††^ | 5.18 | -5.84 | 14.74 | | 0.392 |
|  | Medical treatment requirement | 12.07^††^ | 4.32 | 3.49 | 20.64 | | 0.006 |
| Fetal weight centile >90 (%) ^*^ | Age (years) | 1.06 | 0.06 | 0.95 | 1.18 | | 0.298 |
|  | Ethnicity (low/high risk) | 1.21 | 0.73 | 0.37 | 3.92 | | 0.751 |
|  | Prepregnancy BMI (kg/m^2^) | 1.14 | 0.06 | 1.03 | 1.25 | | 0.009 |
|  | Gestational weight gain (kg) | 1.07 | 0.04 | 0.99 | 1.16 | | 0.073 |
|  | Gestational weight gain (kg) until the 1^st^ GDM visit | 1.08 | 0.06 | 0.97 | 1.19 | | 0.159 |
|  | Excessive weight gain until the 1^st^ GDM visit^†^ | 1.18 | 0.83 | 0.30 | 4.67 | | 0.811 |
|  | Fasting oGTT glucose (mmol/L) | 1.28 | 0.65 | 0.47 | 3.45 | | 0.633 |
|  | 1-h oGTT glucose (mmol/L) | 1.29 | 0.23 | 0.91 | 1.82 | | 0.150 |
|  | 2-h oGTT glucose (mmol/L) | 0.95 | 0.17 | 0.68 | 1.34 | | 0.769 |
|  | HbA1c at the 1st GDM visit (%/mmol/mol) | 1.70 | 1.17 | 0.45 | 6.54 | | 0.435 |
|  | Medical treatment requirement | 2.50 | 1.73 | 0.64 | 9.72 | | 0.186 |
| Fetal abdominal circumference centile (%) ^*^ | Age (years) | 0.65^††^ | 0.61 | -0.55 | 1.86 | | 0.286 |
|  | Ethnicity (low/high risk) | 2.81^††^ | 6.60 | -10.30 | 15.92 | | 0.672 |
|  | Prepregnancy BMI (kg/m^2^) | 1.56^††^ | 0.54 | 0.48 | 2.64 | | 0.005 |
|  | Gestational weight gain (kg) | 0.51^††^ | 0.46 | -0.40 | 1.43 | | 0.269 |
|  | Gestational weight gain (kg) until the 1^st^ GDM visit | 0.78^††^ | 0.57 | -0.36 | 1.91 | | 0.177 |
|  | Excessive weight gain until the 1^st^ GDM visit^†^ | 8.89^††^ | 7.51 | -6.04 | 23.81 | | 0.240 |
|  | Fasting oGTT glucose (mmol/L) | -0.53^††^ | 6.09 | -12.63 | 11.57 | | 0.931 |
|  | 1-h oGTT glucose (mmol/L) | 2.42^††^ | 1.85 | -1.26 | 6.10 | | 0.194 |
|  | 2-h  oGTT glucose (mmol/L) | -0.98^††^ | 1.95 | -4.87 | 2.91 | | 0.618 |
|  | HbA1c at the 1st GDM visit (%/mmol/mol) | 6.74^††^ | 7.68 | -8.52 | 22.00 | | 0.383 |
|  | Medical treatment requirement | 13.58^††^ | 6.54 | 0.58 | 26.58 | | 0.041 |
| Fetal abdominal circumference centile >90 (%) ^*^ | Age (years) | 1.05 | 0.05 | 0.97 | 1.15 | | 0.230 |
|  | Ethnicity (low/high risk) | 1.01 | 0.47 | 0.41 | 2.52 | | 0.980 |
|  | Prepregnancy BMI (kg/m^2^) | 1.05 | 0.04 | 0.98 | 1.14 | | 0.175 |
|  | Gestational weight gain (kg) | 1.06 | 0.04 | 0.99 | 1.13 | | 0.074 |
|  | Gestational weight gain (kg) until the 1^st^ GDM visit | 1.09 | 0.05 | 1.00 | 1.19 | | 0.050 |
|  | Excessive weight gain until the 1^st^ GDM visit^†^ | 2.19 | 1.32 | 0.67 | 7.15 | | 0.196 |
|  | Fasting oGTT glucose (mmol/L) | 0.94 | 0.39 | 0.41 | 2.12 | | 0.877 |
|  | 1-h oGTT glucose (mmol/L) | 1.17 | 0.16 | 0.90 | 1.54 | | 0.243 |
|  | 2-h oGTT glucose (mmol/L) | 0.85 | 0.12 | 0.63 | 1.13 | | 0.259 |
|  | HbA1c at the 1^st^ GDM visit (%/mmol/mol) | 1.46 | 0.80 | 0.50 | 4.25 | | 0.492 |
|  | Medical treatment requirement | 2.78 | 1.47 | 0.98 | 7.85 | | 0.054 |
| Fetal abdominal circumference centile <10 (%) ^*^ | Age (years) | 0.94 | 0.06 | 0.82 | 1.07 | | 0.358 |
|  | Ethnicity (low/high risk) | 0.43 | 0.36 | 0.08 | 2.25 | | 0.317 |
|  | Prepregnancy BMI (kg/m^2^) | 0.90 | 0.07 | 0.77 | 1.06 | | 0.195 |
|  | Gestational weight gain (kg) | 1.01 | 0.05 | 0.92 | 1.12 | | 0.775 |
|  | Gestational weight gain (kg) until the 1^st^ GDM visit | 1.01 | 0.07 | 0.88 | 1.16 | | 0.873 |
|  | Excessive weight gain until the 1^st^ GDM visit^†^ | 0.94 | 0.80 | 0.18 | 5.01 | | 0.943 |
|  | Fasting oGTT glucose (mmol/L) | 0.81 | 0.56 | 0.21 | 3.17 | | 0.762 |
|  | 1-h oGTT glucose (mmol/L) | 0.74 | 0.19 | 0.45 | 1.20 | | 0.222 |
|  | 2-h oGTT glucose (mmol/L) | 0.88 | 0.22 | 0.55 | 1.43 | | 0.611 |
|  | HbA1c at the 1st GDM visit (%/mmol/mol) | 0.38 | 0.36 | 0.06 | 2.39 | | 0.305 |
|  | Medical treatment requirement | 0.38 | 0.36 | 0.06 | 2.39 | | 0.300 |

Linear and logistic regression analyses, adjusted for gestational age

Abbreviations: OR odds ratio, CI confidence interval, BMI body mass index, GDM gestational diabetes mellitus, oGTT oral glucose tolerance test, HbA1c glycated hemoglobin.

* adjusted for gestational age using the Intergrowth 21st fetal size application tool [30]

† according to the Institute of Medicine Guidelines 2009 [28]

†† this value corresponds to a beta-coefficient
