# Supplementary material for: Sex-dependent influence of maternal predictors on fetal anthropometry in pregnancies with gestational diabetes mellitus
Source: BMC Pregnancy Childbirth. 2022 Jun 1;22:460. doi: 10.1186/s12884-022-04767-z (PMC9158189; doi:10.1186/s12884-022-04767-z)
Supplement: Supplementary file 3 — Additional file 3. [file 12884_2022_4767_MOESM3_ESM.docx]

**Additional file 3**

**Table A3.** Risk stratification for fetal weight centile > 90% and fetal abdominal circumference centile > 90% during the 3^rd^ trimester based on prepregnancy BMI and EWG at the 1st GDM visit

| Prepregnancy BMI (kg/m^2^) | EWG at the 1st GDM visit ^*^ | Fetal weight centile >90% ^†^ | Fetal abdominal circumference centile > 90% ^†^ |
| --- | --- | --- | --- |
| < 25 | no | 8% | 14% |
| < 25 | yes | 13% | 24% |
| ≥ 25 | no | 15% | 23% |
| ≥ 25 | yes | 24% | 37% |
|  |  |  |  |

Probability analyses using logistic regression models.

Abbreviations: BMI body mass index, EWG excess weight gain, GDM gestational diabetes mellitus.

* according to the Institute of Medicine Guidelines 2009 [28] †

† adjusted for gestational age using the Intergrowth 21st fetal size application tool [30]
